# Development and validation of a prediction model estimating the 10-year risk for type 2 diabetes in China

**DOI:** 10.1371/journal.pone.0237936

**Published:** 2020-09-03

**Authors:** Xian Shao, Yao Wang, Shuai Huang, Hongyan Liu, Saijun Zhou, Rui Zhang, Pei Yu

**Affiliations:** NHC Key Laboratory of Hormones and Development, Tianjin Key Laboratory of Metabolic Diseases, Chu Hsien-I Memorial Hospital & Tianjin Institute of Endocrinology, Tianjin Medical University, Tianjin, China; Shanghai Diabetes Institute, CHINA

## Abstract

**Purpose:**

To derive and validate a concise prediction model estimating the 10-year risk for type 2 diabetes (T2DM) in China.

**Methods:**

A total of 11494 subjects from the China Health and Nutrition Survey recorded from 2004 to 2015 were analyzed and only 6023 participants were enrolled in this study. Four logistic models were analyzed using the derivation cohort. Methods of calibration and discrimination were used for the validation cohort.

**Results:**

In the derivation cohort, 257 patients were identified from a total of 4498 cases. In the validation cohort, 92 patients were identified from a total of 1525 cases. Four models performed nicely for both calibration and discrimination. The AUC in the derivation cohort for models A, B, C and D were 0.788 (0.761–0.816), 0.807 (0.780–0.834), 0.905 (0.879–0.932) and 0.882 (0.853–0.912), respectively. The Youden index for models A, B, C and D were 1.46, 1.48, 1.67 and 1.65, respectively. Model C showed the highest sensitivity and model D showed the highest specificity.

**Conclusion:**

Models A and B were non-invasive and can be used to identify high-risk patients for broad screening. Models C and D may be used to provide more accurate assessments of diabetes risk. Furthermore, model C showed the best performance for predicting T2DM risk and identifying individuals who are in need of interventions, current approach improvement and additional follow-up.

## Introduction

The global prevalence of adult diabetes is increasing and becoming a major public health problem. In 2017, The International Diabetes Federation (IDF) estimated the global diabetes prevalence as 425 million among adults aged 20–79 years of age (8.8%) and over 224 million adults were found to be living with undiagnosed diabetes. A higher proportion of undiagnosed diabetes cases were found in low- and middle-income countries. Moreover, over one third (36.5%) of deaths attributed to diabetes occurred in people under the age of 60 years [1]. In China, the true prevalence of undiagnosed diabetes may be underestimated. A total of 9.7%-10.9% of the population was diagnosed with diabetes and 35.7%-60.7% were cases of undiagnosed diabetes [2–4]. Type 2 diabetes (T2DM) and its associated complications have caused significant economic burden to patients and is a major public health challenge facing China [5, 6]. Thus, the prevention and early management of diabetes and its complications are necessary this burden for the general Chinese population. Risk prediction models have considerable potential to help diagnose a patient. During the past 20 years, dozens of prediction models for diabetes have been developed. However, none of these models have been routinely used in China thus far.

Clinical utility for imperfect prediction models has been a concern. Risk scores derived from Caucasian populations may not be suitable for Chinese populations as there is significant geographical and biological variation in China. There have been many types of T2DM risk prediction scores and models generated in China [7–16], but they all face several limitations. Most do not account for lifestyle variations, such as physical activity, dietary behavior or sleep duration. Others are based on invasive and cost-effective data such as blood tests and radiology or on a small and inappropriate selection of the cohort. Others are based on a short-term follow-up or lack transparent reporting of the steps deriving the model.

The aims of this article were to derive large population-based, innovative and simple models for screening high-risk non-diabetic individuals in China using available data. We also assess the clinical utility of four algorithms using decision curve analysis. In addition, this study also compared the performance of models developed to evaluate their effectiveness.

## Methods

This cohort study complies with the Prognosis Research Strategy (PROGRESS) framework. Transparent Reporting of a multivariable prediction model for Individual Prognosis or Diagnosis (TRIPOD) statement was used in this study.

### Data and participants

Data was downloaded from the China Health and Nutrition Survey on 2019/1/9 (https://www.cpc.unc.edu/projects/china/). The survey was performed within a period of 7 days using a multi-stage, random clustering method, and selected samples from 15 provinces and cities in China. A total of 11494 subjects aged 20–80 years were observed from 2004 to 2015.

**Fig 1:**
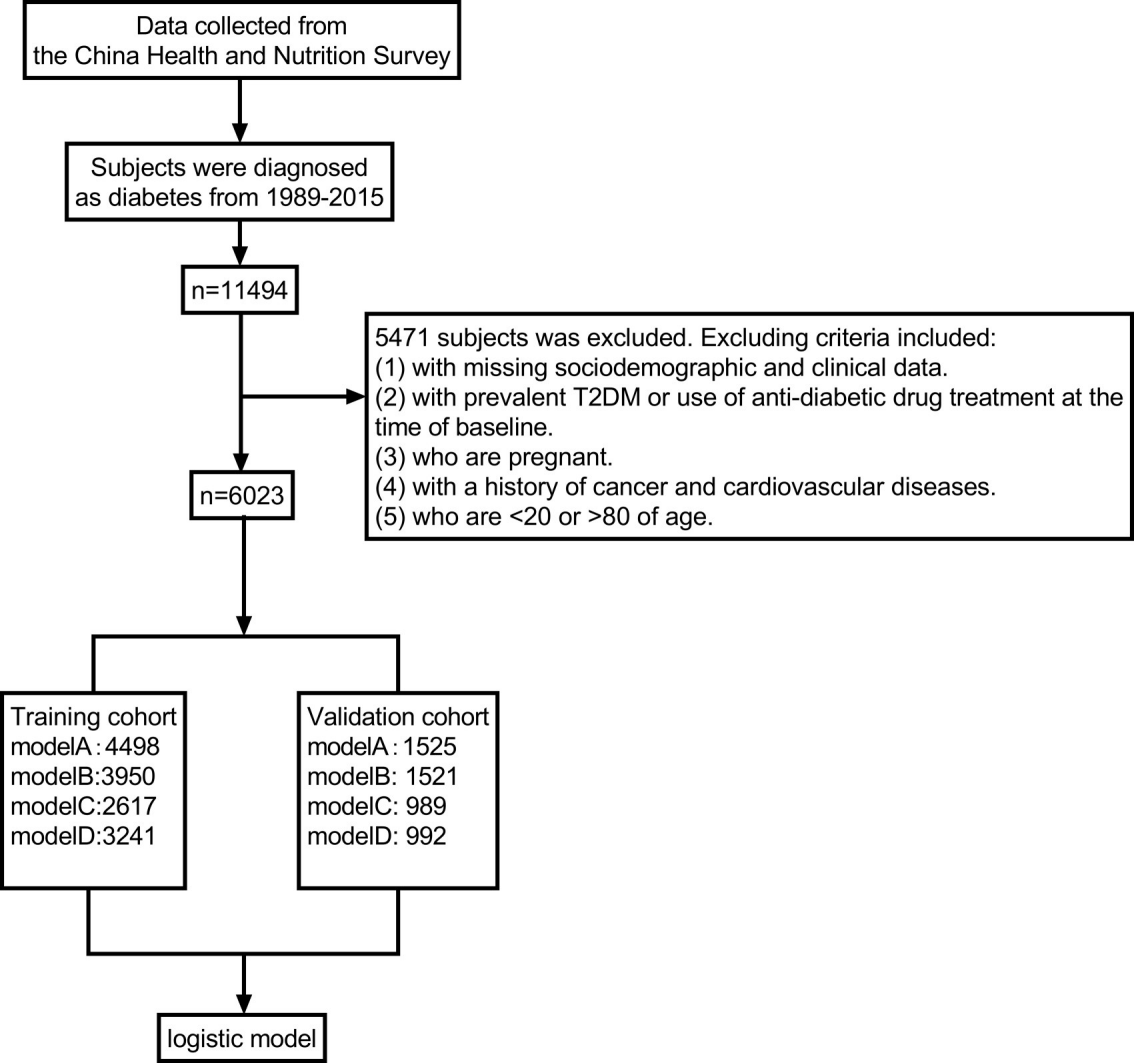
Flow diagram schematic of the study performed.

A flow diagram of the study performed is summarized in Fig 1. Patient exclusion criteria excluding included individuals (1) with missing sociodemographic and clinical data, (2) with prevalent T2DM or use of anti-diabetic drug treatment at the time of baseline, (3) who are pregnant, (4) with a history of cancer and cardiovascular diseases and (5) who are <20 or >80 of age. A total of 5453 participants were excluded and 6023 subjects were included in the final analysis.

A total of 8 provinces were selected and about three-fourths of participants (N = 4498) were added into a training data set. The remaining 2 provinces were included in the outside validation set (N = 1525) using a random sampling method.

### Outcomes

According to the ADA 2015 criteria [17], meeting any of the following conditions during the follow-up period can be defined as T2DM, including (1) use of any anti-diabetic medications or insulin, (2) record of diabetes in the survey, (3) a FPG ≥ 7.0 mmol/L, or 2 h-PG ≥ 11.1 mmol/L, or HbA1c ≥ 6.5%, or random glucose ≥11.1 mmol/L.

### Variable measurements

According to research on risk factors associated with diabetes, demographic data (age, gender, ethnicity), personal history (education level, smoking status, alcohol intake, physical activity, history of cardiovascular or cerebrovascular accidents (CVD), history of hypertension), physical characteristics (body mass index (BMI), weight, height, systolic and diastolic blood pressure (SBP and DBP), waist circumference, triceps skin fold thickness, hours of sleep), dietary intake (soft drink, tea, coffee, calories, fat, protein, carbohydrates (average consumption of three days)) and laboratory parameters (fasting plasma glucose (FPG), hemoglobin A1c (HbA1c), triglycerides (TGs), total cholesterol (TC), high-density lipoprotein (HDL-c), low-density lipoprotein (LDL-c), insulin) were measured. Previous history and demographic characteristics were obtained through a standard questionnaire. Detailed household dietary intake information for three consecutive days was collected. History of CVD was defined as previous ischemic heart disease and/or cerebrovascular incidents. BMI was calculated as weight (kg)/height (m^2^). Height was measured to the nearest 0.5 cm and weight was measured to the nearest 0.1 kg. Blood pressure was measured in the right arm three times and averaged using a mercury sphygmomanometer. After overnight fasting at least 10 h, blood samples were collected in the morning and were processed within 2 h.

### Sample size

The rule of thumb for fitting multivariate models suggests that each variable (EPV) requires 10 events to avoid overfitting. For models A, B, C and D, we finally chose 8, 17, 20 and 5 predictors variables, respectively. The sample sizes for models A, B, C and D were 80, 170, 200 and 50, respectively. We found that our sample size completely met this rule.

### Data preparing

Data preparation included processing missing data, selection of variables and defining and balancing the training and validation data sets. Individuals with missing outcomes, sociodemographic or clinical values were excluded from our analysis. Univariable and multivariable binary logistic regression as well as least absolute shrinkage and selection operator (LASSO) regression were used for variable selection. Significance for univariable and multivariable binary logistic regression was set at P < 0.05. From this, the relationship between each variable with T2DM could be identified. LASSO regression chose potential predictors by choosing minimum λ criteria, as the area under the receiver operating characteristic (AUC) value plotted versus log (λ). In addition, penalty function in the LASSO regression helped avoid overfitting and aided in the development of a robust model. A simple random sampling method using a 3: 1 ratio was used to balance the training and validation data sets.

### Risk groups

Age was categorized into 6 groups for each decade including 20–30, 31–40, 41–50, 51–60, 61–70 and 71–80. Nationalities included Han, Mongolian, Hui, Miao, Zhuang, Buyi, Korean, Man, Dong, Tujia and other. Education level was categorized into 6 levels including illiterate, primary school, lower middle school, upper middle school, technical or vocational degree, university or college degree and higher. Physical activities were categorized into 5 groups including very light, light, moderate, heavy or very heavy. Waist circumference was categorized into 6 groups including <60, 61–70, 71–80, 81–90, 91–100 and >100cm. BMI was categorized into 4 groups including <18.5, 18.6–23, 23.1–28 and >28 kg/m^2^. Sleep hours were categorized into 4 groups including 1–4, 5–8, 9–12 and >12h. Triceps skin fold thickness, LDL, HDL, TC and TG were categorized into 6 groups according to population-based 5th, 25th, 50th, 75th and 95th percentiles. Calories were categorized into 6 groups including <800, 800–1500, 1501–2500, 2501–3500, 3501–4500 and >4500kcal per day. Carbohydrate intake was categorized into 5 groups including <150, 150–300, 301–450, 451–600 and >60g per day. Protein was categorized into 5 groups including <25, 26–50, 51–70, 71–90 and >90g per day. Fat was categorized into 6 groups including <25, 26–50, 51–70, 71–90, 91–110 and >110g per day. FPG was categorized into 5 groups including <2.80, 2.81–6.09, 6.10–7, 7.01–10.0 and >10.0mmol/L. HbA1c was categorized into 3 groups including <6.1, 6.1–6.5 and >6.5%. Insulin was categorized into 5 groups including <5, 5–15, 16–25, 26–35 and >35μIU/L.

### Model development and validation

The 6023 participants were divided into a training set (N = 4498) and a validation set (N = 1525) using a simple random sampling method involving a 3: 1 ratio. Data in the training set were used to develop models for undiagnosed diabetes. Univariable and multivariable binary logistic regression and lasso regression were applied to filter risk factors. Nomograms were constructed using the rms package in R software. To achieve an unbiased estimate for the models, the internal validation was performed in the training set using a bootstrap sampling method. Then, external validation was performed using the AUC in the validation set. Differences between different AUCs were compared using the DeLong method. The Youden's index was used to identify the best cut-off value for undiagnosed diabetes. The accuracy of these models was calculated. Clinical usefulness was evaluated using net benefit. Decision curves of the four models were plotted using rmda package in R software (version 3.6.0 http://www.r-project.org).

### Statistical analysis

Continuous variables were described as median (25th–75th percentile) or mean ± SD. Categorical data were presented as number (percentage). The difference between the model derivation cohort and model validation cohort was compared using Student's t tests for continuous data and Chi-squared tests for categorical variables. The Mann-Whitney U and Kruskal-Wallis tests were applied for variables with skewed distributions. The predictive performance of the constructed predictive models was evaluated using accuracy, sensitivity and specificity, as well as receiver operating characteristic (ROC) curves and the AUC value. The models were evaluated by Youden index, accuracy, precision, sensitivity, specificity, optimal cutoff value, positive predictive value (PPV), negative predictive value (NPV), true positive rate (TPR), false positive rate (FPR), false negative rate (FNR), true negative rate (TNR), false discovery rate (FDR) and AUC. For each variable, one category was chosen as the control and odds ratios (ORs) and 95% confidence intervals (CIs) were calculated for other categories. All statistical analyses were performed using R software version 3.6.0 (http://www.r-project.org). R packages aiding in these analyses included rms, rmda, pROC, and shiny, plot. A P <0.05 was considered as statistically significant for all tests.

## Results

### Baseline characteristics of participants

A total of 5471 cases who did not have a complete record were excluded from this analysis. As a result, 6023 subjects met the inclusion criteria and contained data of 10 or more years of follow-up visits. Three quarters of this group (n = 4498) was randomly allocated into the derivation cohort and the remaining (n = 1525) cases were allocated into the validation cohort (Fig 1). Table 1 summarizes the baseline characteristics of study subjects included in both the derivation and validation sets. The mean age of individuals in the derivation cohort was 42.0 ± 18.8 years and a total of 2092 (46.5%) were male. In total, 4498 (67%) cases contained complete information for model A variables, 3950 (59%) for model B, 2617 (39%) for model C and 3241 (48%) for model D. A total of 257 newly diagnosed diabetes cases accounted for 5.7% of the total population from 2004 to 2015 in the derivation cohort. The development and validation cohorts showed similar sociodemographic, physical examinations and laboratory characteristics. Table 1 summarizes medical characteristics for the cases. For the variables of interest in the derivation cohort, the average of the triceps skin fold was 15.0 ± 8.1 cm, average sleep hours were 8.15 ± 1.32 hours, median calorie consumption was 2213.2 kcal, median carbohydrate consumption was 316.4 g, median fat consumption was 68.6 g and median protein consumption was 66.7 g. More details of study participants are shown in Table 1.

**Table 1:** Baseline characteristics of study subjects.

Characteristics	Derivation cohort	Validation cohort	P value
Age, mean (SD, yrs)	42.0 (18.8)	41.1 (17.7)	<0.001***
Gender			
Male	2092 (46.5)	710 (46.6)	0.974
Female	2406 (53.5)	815 (53.4)	
Ethnic Groups			
Han	4244 (94.4)	1282 (84.1)	<0.001***
Mongolian	0	12 (0.8)	
Hui	17 (0.4)	0	
Miao	91 (2.0)	0	
Zhuang	59 (1.3)	0	
Buyi	25 (0.6)	0	
Korean	0	7 (0.5)	
Man	0	217 (14.2)	
Dong	2 (0.04)	0	
Tujia	12 (0.3)	0	
Other	48 (1.1)	7 (0.5)	
Highest Level of Education Attained			<0.001***
None	1026 (22.8)	173 (11.3)	
Grad from primary	1118 (24.9)	425 (27.9)	
Lower middle school degree	1348 (30.0)	503 (33.0)	
Upper middle school degree	644 (14.3)	175 (11.5)	
Technical or vocational degree	236 (5.2)	137 (9.0)	
University or college degree	126 (5.8)	111 (7.3)	
Master's degree or higher	0	1 (0.1)	
Smoking recorded	1421 (31.6)	535 (35.1)	0.011
Alcohol recorded	1435 (31.9)	486 (31.9)	0.971
Coffee	79 (1.8)	14 (0.9)	0.022
Soft drink	1112 (24.7)	309 (20.3)	<0.001***
Tea	1884 (41.9)	331 (21.7)	<0.001***
Hypertension	428 (9.5)	154 (10.1)	0.487
Waist circumference, mean (SD, cm)	81.3 (9.7)	83.0 (9.9)	<0.001***
BMI, mean (SD, kg/m²)	23.2 (3.3)	23.9 (3.5)	<0.001***
Triceps skin fold, mean (SD, mm)	15.0 (8.1)	17.6 (9.0)	<0.001***
Systolic pressure, mean (SD, mmHg)	122.8 (18.2)	124.5 (18.5)	<0.001***
Diastolic pressure, mean (SD, mmHg)	78.5 (11.0)	82.1 (11.8)	<0.001***
Sleep time, mean (SD, hours)	8.15 (1.32)	7.97 (1.50)	<0.001***
Physical activity			0.041
Very light	1019 (22.7)	373 (24.5)	
Light	1139 (25.3)	278 (18.2)	
Moderate	741 (16.5)	185 (12.1)	
Heavy	1485 (33.0)	601 (39.4)	
Very heavy	28 (0.6)	6 (0.4)	
Diet			
Total calories, median (interquartile range, kcal)	2213.2 (1809.0–2675.0)	1959.2 (1571.5–2361.7)	<0.001***
Carbohydrate, median (interquartile range, g)	316.4 (264.9–391.0)	281.5 (226.4–339.9)	<0.001***
Fat, mean (interquartile range, g)	68.6 (47.0–94.1)	57.9 (39.4–83.3)	<0.001***
Protein, mean (interquartile range, g)	66.7 (53.4–82.1)	56.3 (43.7–71.3)	<0.001***
Blood test			
HDL, mean (SD, mmol/L)	1.5 (0.4)	1.4 (0.4)	<0.001***
LDL, mean (SD, mmol/L)	3.1 (1.0)	3.0 (0.9)	<0.001***
Insulin, median (interquartile range, μIU/L)	10.2 (7.3–14.8)	9.7 (6.7–15.0)	<0.001***
HbA1c, mean (SD, %)	5.7 (0.9)	5.7 (1.0)	<0.001***
Glucose, mean (SD, mmol/L)	5.5 (1.5)	5.4 (1.6)	<0.001***
TG, mean (SD, mmol/L)	3.4 (1.3)	1.8 (1.6)	<0.001***
TC, mean (SD, mmol/L)	5.0 (1.0)	4.9 (1.0)	<0.001***
Complete data for model A	4498 (0.67)	1525 (0.77)	
Complete data for model B	3950 (0.59)	1521 (0.76)	
Complete data for model C	2617 (0.39)	989 (0.50)	
Complete data for model D	3241 (0.48)	992 (0.50)	

Data are presented as median (interquartile range), mean (SD), number (%).

### Selection of features for model development

**Fig 2:**
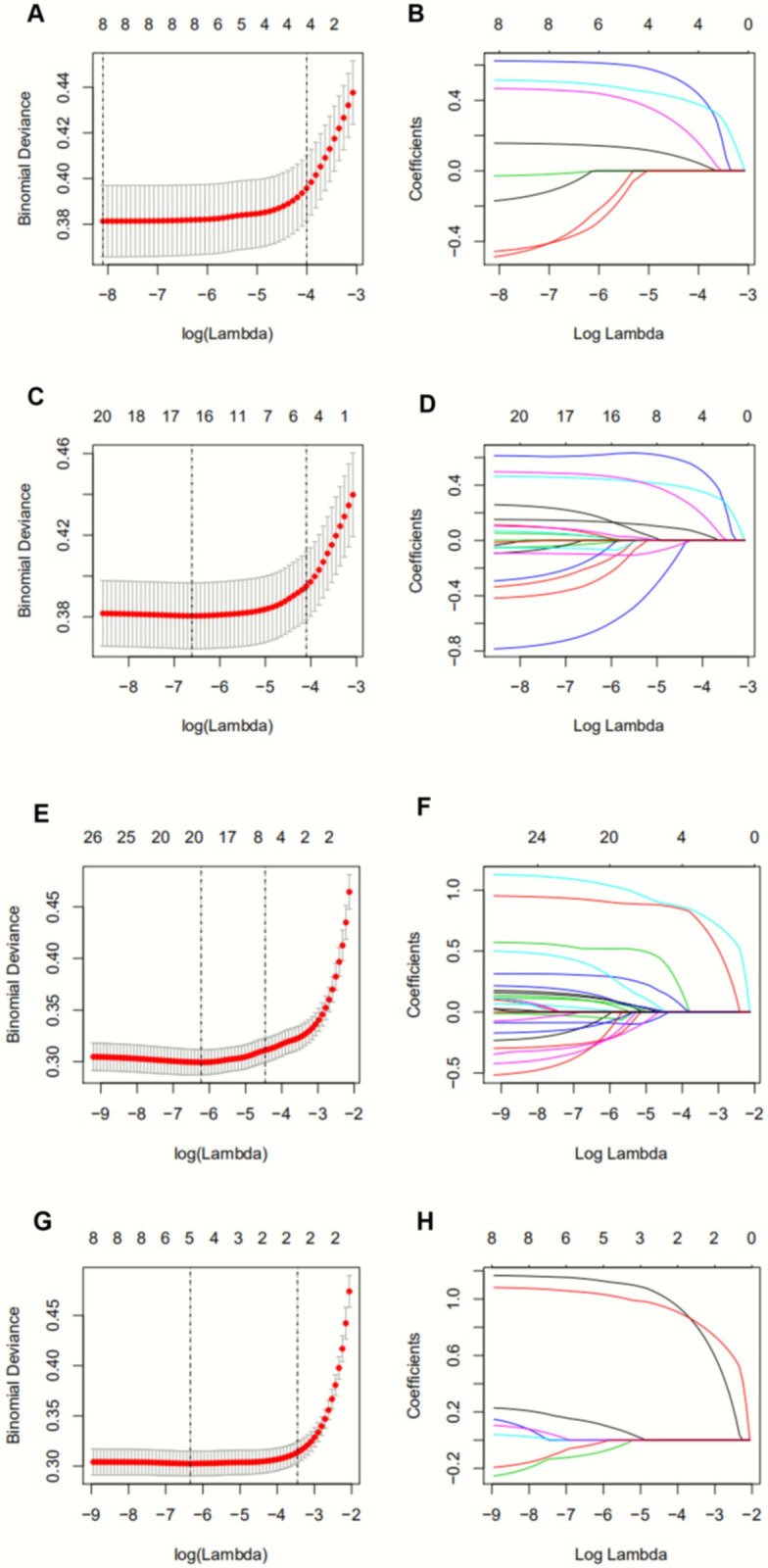
Feature selection using the least absolute shrinkage and selection operator (LASSO) binary logistic regression model. (A) Turning parameter (λ) selection in the LASSO model used 10-fold cross-validation via minimum criteria. The AUC curve was plotted versus log (λ). Dotted vertical lines were generated at the optimal values using the minimum criteria (the 1-SE criteria). A λ value of 0.0003 with log (λ), -8.099 was chosen (1-SE criteria). (B) LASSO coefficient profiles of the 8 features. A coefficient plot was generated against the log (λ) sequence. (C) Features that were selected for the second model. Similarly, dotted vertical lines were drawn at optimal values and λ value of 0.001 with log (λ) -6.520 was chosen. (D) LASSO coefficient profiles of the 17 features are shown. A coefficient plot was generated against the log (λ) sequence. (E) A list of features selected for the third model. A λ value of 0.002 with a log (λ) of -6.227 was chosen. (F) A list of LASSO coefficient profiles of the 20 features. (G) A list of features selected for the fourth model. A λ value of 0.001 with a log (λ) of -6.526 was chosen. (H) LASSO coefficient profiles of the 5 features are listed.

S1 Table shows univariable and multivariable logistic regression analyses. Analyses revealed that nationality, highest level of education, alcohol intake, soft drink and tea ingestion, hypertension, BMI, waist circumference, triceps skin fold, SBP, DBP, physical activity, carbohydrates, fat or protein intake, LDL, HDL, TC, TG, insulin, HbA1c and fasting blood glucose levels were potential risk factors for newly diagnosed diabetes. S2 Table shows the LASSO regression of risk factors for the derivation cohort. Of all features, 8 were selected as potential predictors for the derivation cohort for model A (Fig 2A and 2B). A total of 21 features were reduced to 17 potential predictors in the cohort for model B (Fig 2C and 2D). A total of 27 features were reduced to 20 potential predictors in the cohort for model C (Fig 2E and 2F). A total of 6 features were reduced to 5 potential predictors in the cohort for model D (Fig 2H and 2K). We finally chose 8, 17, 20 and 5 predictors from 8, 21, 27, 6 primary variables for developing models A, B, C and D, respectively. In addition, there were no features observed with zero coefficients in the LASSO logistic regression model. The coefficients of all features are listed in S2 Table.

### Development of the logistic individualized prediction model

**Fig 3:**
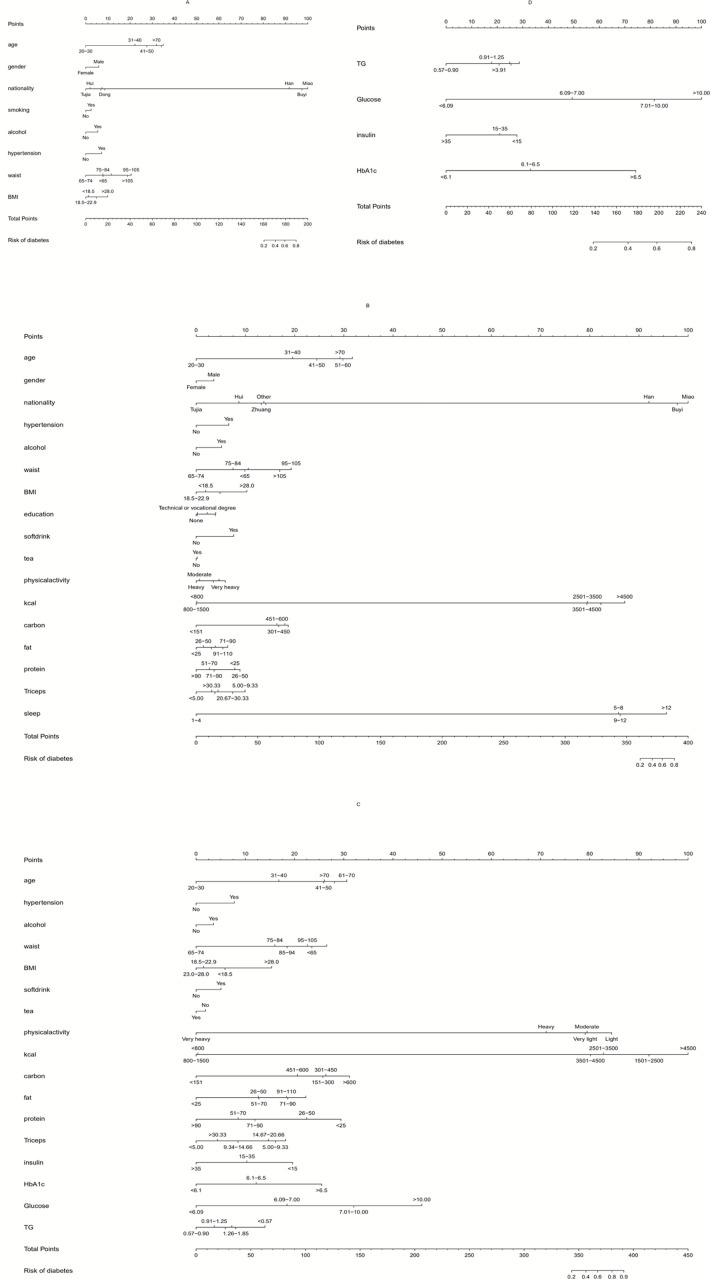
Nomogram for models A, B, C and D.

As shown in Fig 3A–3D, the nomogram of the logistic model A was a quantitative and convenient tool that predicts the risk of T2DM using age, gender, ethnicity, hypertension, smoking, alcohol intake, waist and BMI in the training cohort (S2 Table). The nomogram of logistic model B included most variables in model A plus levels of education, soft drink and tea consumption, physical activity, calories, carbohydrates, fat, protein, triceps skin fold thickness and sleep hours. The nomogram of logistic model C included variables in model B plus LDL, HDL, TC, TGs, insulin, FBG and HbA1c levels. The nomogram of logistic model D included LDL, HDL, TC, TGs, insulin, FBG and HbA1c levels. To obtain a personalized 10-year risk of T2DM, a vertical line was drawn from the value on the point scale to evaluate the points, then the points were added together to obtain each variable value. The sum included total points and was matched to risk on the bottom axis.

### Model discrimination

**Fig 4:**
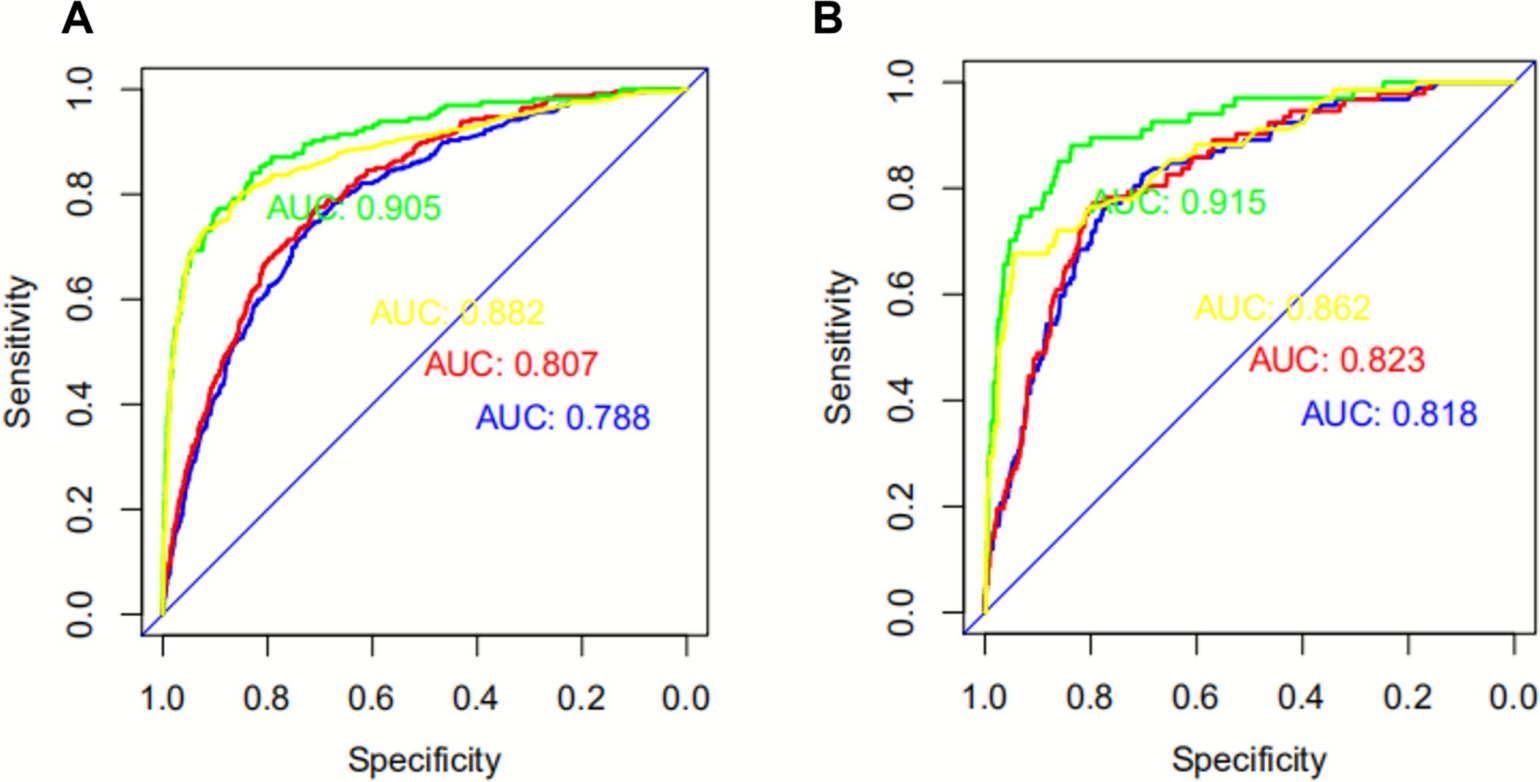
ROC curves of the nomogram for the 10-year T2DM risk in the training and validation cohorts. (A) ROC curves of logistic regression models for 10-year T2DM risk in the training cohort. The AUC of model A was 0.788 (0.761–0.816), the AUC of model B was 0.807 (0.780–0.834), the AUC of model C was 0.905 (0.879–0.932) and the AUC of model D was 0.882 (0.853–0.912). (B) The ROC curves of the logistic regression models for the 10-year T2DM risk in the validation cohort. The corresponding AUC of models A, B, C and D were 0.818 (0.775–0.861), 0.823 (0.780–0.865), 0.915 (0.877–0.953) and 0.862 (0.813–0.912), respectively. ROC: receiver operating characteristics curves, AUC: area under the curve. *Using bootstrap resampling (times = 1000).

S3 Table shows the performance of each model in the training and validation sets. The resulting model was internally validated by 2000 bootstrap resampling. All models showed good calibration and discrimination. ROC curves are shown in Fig 4A and 4B. Model C showed the best overall performance, followed by model D. In the training cohort of model A, the AUC was 0.788 (0.761–0.816), the Youden index was 1.46, the sensitivity and specificity rates were 74.71% and 71.16%, respectively. The corresponding values for model A in the validation cohort were 0.818 (0.775–0.861), 1.54, 77.17% and 76.91%. The corresponding values for model B in the training cohort were 0.804 (0.776–0.831), 1.48, 77.53% and 70.83%. The corresponding values for model B in the validation cohort were 0.823 (0.780–0.865), 1.57, 77.17% and 79.65%. In the training cohort of model C, the corresponding values were 0.904 (0.877–0.931), 1.67, 84.05% and 83.01%. The corresponding values for model C in the validation cohort were 0.915 (0.877–0.953), 1.72, 88.06% and 83.73%. In the training cohort of model D, the corresponding values were 0.885 (0.857–0.913), 1.65, 73.08% and 92.25%. The corresponding values for model D in the validation cohort were 0.862 (0.813–0.912), 1.62, 67.65% and 94.59%.

### Model calibration

**Fig 5:**
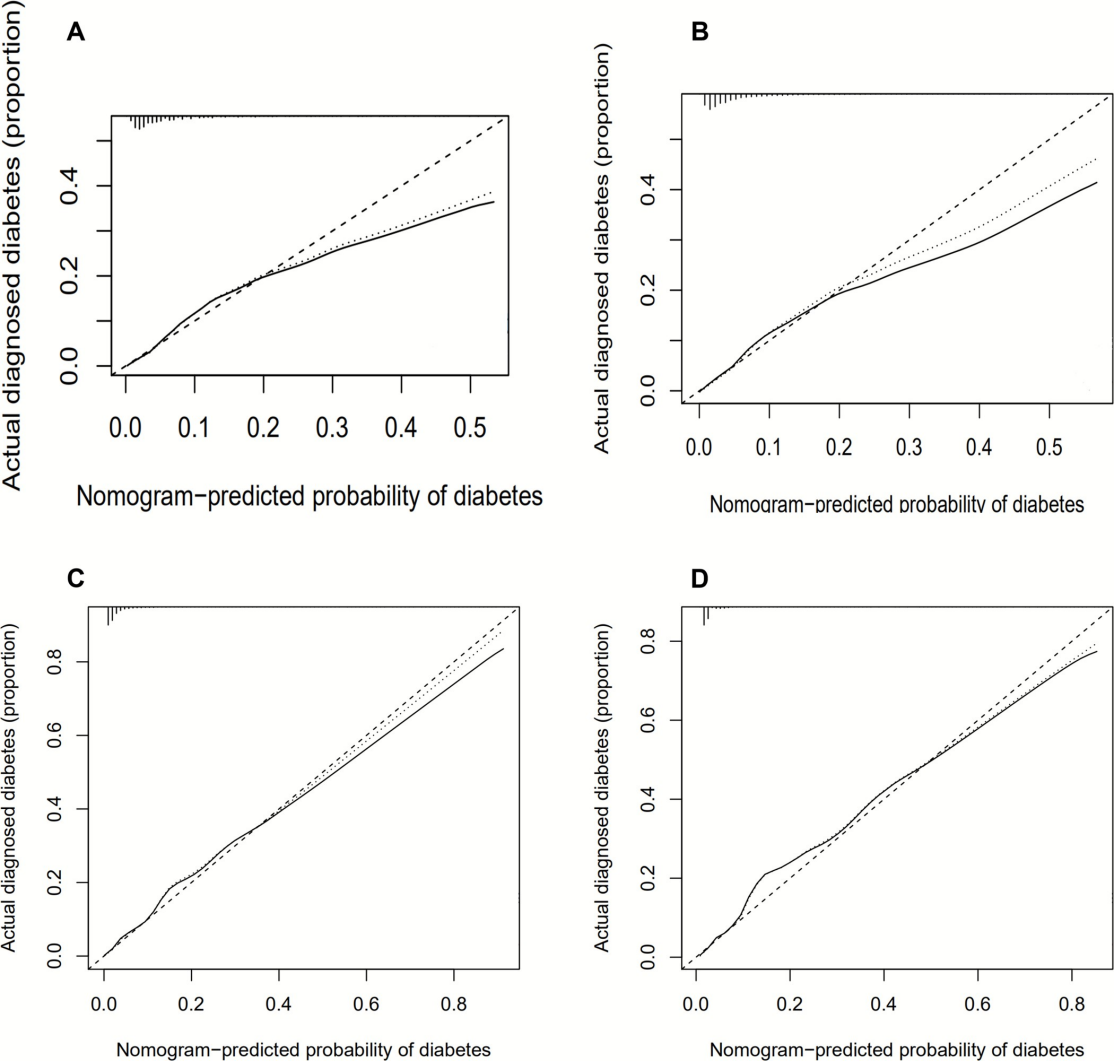
Calibrations for nomograms of logistic regression models using the bootstrap sampling method (B = 1000 repetitions). Calibration curves for nomograms of (A) model A. (B) model B. (C) model C and (D) model D.

The mean absolute error of models A and B were 0.006 and the mean absolute error of models C and D were 0.004. Internal bootstrap validation showed that the nomogram of the model A derived curve was close to the bias-corrected curve and the ideal curve at a probability between 0 and 0.20. When the probability was lower than 0.20, model A may underestimate the probability of undiagnosed diabetes (Fig 5A). Model B was similar, where the start point of underestimation was also 0.20 (Fig 5B). The nomogram of the model C derived curve performed well on all scales. Model D resembled model C (Fig 5C and 5D). Models C and D fitted well and showed good calibration.

### Decision curve analysis

**Fig 6:**
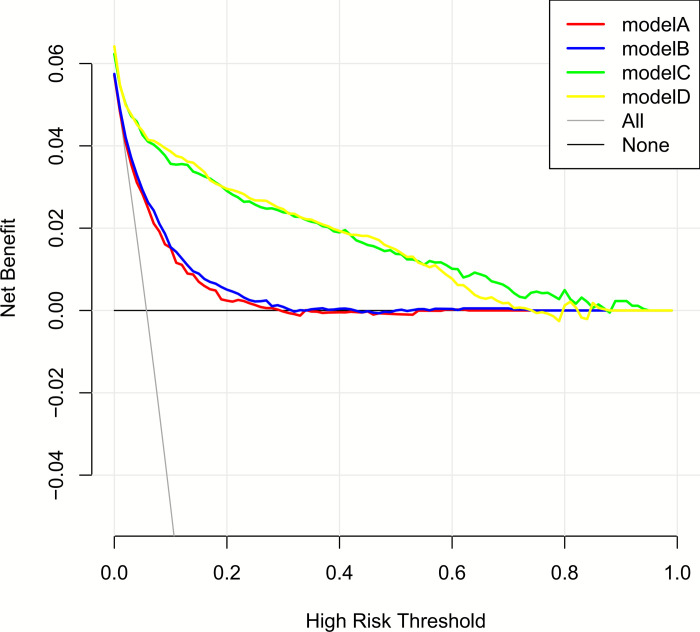
Net benefit curves for models A, B, C and D.

To compare clinical usefulness of the models, decision curve analysis was performed as shown in Fig 6. On the y axis, the vertical distance to the x axis showed the standard net benefit. The x axis showed the threshold probability for diabetes. Each line represented clinical usefulness for each model. In our analysis, models A, B, C and D all demonstrated better cost-effectiveness than no treatment. Models C and D exhibited the best performance. Models A and B showed slightly improved net benefit compared to models C and D. Compared to strategies that either no or all patients received intervention, models C and D showed higher net benefit. When absolute risk threshold was approximately 60%, these interventions were shown to be useful.

## Discussion

In this study, we developed and validated four models to predict the 10-year risk of T2DM in Chinese residents. A total of four models were produced. Both internal and external validation was performed in the cohort and the results showed good performance in discrimination and calibration for the four models. Net benefit curves also demonstrated the clinical benefit of this nomogram. Results showed that although model B did not precede model A significantly, the other two models showed considerable improvement (S3 and S4 Tables), with the best overall performance being shown for model C. Model C showed the best discrimination and highest sensitivity. Even though model D, which only included blood test results, showed better discrimination than models A and B, it had the lowest sensitivity and highest specificity (S3 Table). Model C, based on comprehensive details in lifestyle and clinical results, could be used to provide a multi-aspect management for pre-diabetic or diabetic patients and provide better information regarding potential effects of risk factors. For those who have been diagnosed with diabetes, using nomograms for self-monitoring could delay the occurrence and progress of complications. These models improve current guidance using fixed thresholds for fasting blood glucose or HBA1c levels as diagnostic criterion for diabetes, as it contains subjects with high-risk for T2DM and not only individuals already diagnosed with T2DM. Model C showed the highest clinical net benefit revealed by the DCA curve in Fig 5, meaning that model C had the greatest clinical use.

In addition, models A and B with basic and non-invasive prediction factors could be used to identify high-risk diabetes cases that require a test for fasting insulin, blood glucose or HBA1c levels. Patients could perform these tests independently in their homes. Model A was simpler than model B and included 7 basic common risk factors, such as age and gender. Although model B was not superior to model A regarding performance of ROC curve, predictors such as sleep duration and physical activity were more important and controllable. Model B included a more comprehensive detail of lifestyle and could be used for self-management and medical treatment advising. After identifying patients at high risk by using model B, patients need to complete relevant tests including blood lipid, insulin, FBG and HbA1c level analyses. Models C and D may be used to provide more accurate assessments of diabetes risk and individualized blood glucose management. Overall, the use of these models is more accurate in predicting diabetes risk and is also suitable for extensive diabetes screening and self-monitoring.

In China, there are many T2DM risk prediction scores or models generated [7–16], Common risk factors included age, sex, ethnicity, waist circumference, BMI and hypertension. Although these unmodifiable risk factors played roles in T2DM, changes in modifiable factors such as dietary behavior can reduce the risk and influence disease progression [18]. Recent meta-analysis also revealed an association between dietary behaviors and physical activity in relation to T2DM [19–22]. In addition, meta-analysis demonstrated a U-shaped relationship between sleep duration and T2DM risk, with the lowest risk being 7–8 h of sleep per day [23]. This result is consistent with our findings. In our study, we evaluated beverages, physical activity, calorie intake, carbohydrates, fat, protein and sleep duration to establish a risk model emphasizing the impact of lifestyle on T2DM development and progression. There was slight improvement when model B included these variables that may be attributed to the limited number of study subjects. As known, published models in China have not been routinely used in the clinical. QDiabetes-2018 is a successful example of a risk model put into clinical use [24]. Of the models, seven were from one region [8–10, 12, 13, 15, 16] and two were from multicenter in China [11, 14]. In our study, the China Health and Nutritional survey was conducted in 10 provinces including the municipalities of Beijing and Shanghai from 1989 to 2015. Five used nomograms [13–16, 25]. And five used risk score [8–12]. Compared to risk score, a nomogram is more user friendly and accurate based on continuous variables and simple algorithm diagrams. One nomogram used data from an abdominal CT [15], however, the costs of a CT make it unsuitable for screening and extensive use. K. Wang *et al*. established their nomogram using semi-lab indicators [16], however, there is a lack of medical examinations routinely performed in China. In addition, the AUCs of the nomogram in women were unsatisfactory. In models A and B, it is not consuming to finish the prediction and the AUCs remain satisfactory. Furthermore, these nomograms [13, 15, 16] did not have sufficient calibration to provide evidence on predicted probability in accordance with actual observed probability. In our model, all risk models were internally validated using the bootstrap sampling method and externally validated in the validation set.

Limitations do exist in this study. First, only 6023 patients contained complete data. There was a significant difference between most characteristics for included and excluded participants (S5 Table). Age was set to be over 20, contributing to a significant difference. According to the excluding criterion, individuals with missing sociodemographic and clinical data needed to be removed from the study and these individuals accounted for a large proportion of subjects. Thus, additional more external validation of these models and more complete data are needed before clinical use. Second, there may be under-ascertainment of T2DM diagnosis since record terms were used as a criterion and we did not have complete data on oral glucose tolerance testing (OGTT) tests. This may lead to misclassification bias for outcomes. Only 257 newly diagnosed diabetes cases from 2004 to 2015 were included in the derivation cohort and overfitting may be difficult to avoid. Our derivation cohort contained 2617 events, the recommended events were at least 10 and there were on average 131 events per variable predictor. Split sample validation is still valuable in this study. Validation has been completed by randomly selecting individuals from 2 provinces in China to develop the score. Furthermore, emergency algorithms, such as machine learning, neural networks and decision trees can be used to build a risk model.

## Conclusions

Model A can be completed at home and patients can decide whether to pursue further blood testing. Model B includes more comprehensive details regarding lifestyle and can be used for self-management and to provide advice when seeking medical treatment. Model C showed the best performance and can identify patients who need more interventions and intensive follow-ups. Furthermore, it can be used to develop an individualized intervention plan. If these models were used in the clinic, such as in medical electronic record system and self-management systems, there would be a decrease in economic burden associated with diabetes and better management of complications associated with this malady.

## Supporting information

S1 Table Logistic regression and cox regression analysis in the derivation cohort. (DOCX)

S2 Table Feature selection using the least absolute shrinkage and selection operator (LASSO) model binary logistic regression model. (DOCX)

S3 Table Prediction performance of the nomogram for estimating the 10-year risk of T2DM. (DOCX)

S4 Table Delong comparison between different logistic models. (DOCX)

S5 Table Baseline characters of the participants inclued and excluded. Data are presented as median (interquartile range), mean (SD), number (%).
(DOCX)
