# Supplementary material for: Development and validation of a prediction model estimating the 10-year risk for type 2 diabetes in China
Source: PLoS One. 2020 Sep 3;15(9):e0237936. doi: 10.1371/journal.pone.0237936 (PMC7470416; doi:10.1371/journal.pone.0237936)
Supplement: S1 Table — (DOCX) [file pone.0237936.s001.docx]

| S1 Table. Logistic regression and cox regression analysis in the derivation cohort | | | | | | | |
| --- | --- | --- | --- | --- | --- | --- | --- |
|  | Univariate logistic regression | | |  | Multivariate logistic regression | | |
|  | β-Coefficient | OR (95%CI) | P-Value |  | β-Coefficient | OR (95%CI) | P-Value |
| Variables |  |  |  |  |  |  |  |
| Age (years) | 0.003 | 1.003 (0.996- 1.010) | 0.384 |  |  |  |  |
| Gender |  |  |  |  |  |  |  |
| Male | - | 1.000 | - |  |  |  |  |
| Female | -0.041 | 0.960 (0.746-1.236) | 0.750 |  |  |  |  |
| Ethnic Groups |  |  |  |  |  |  |  |
| Han | - | 1.000 | - |  | - | 1.000 | - |
| Mongolian | NA | NA | NA |  |  |  |  |
| Hui | NA | NA | NA |  |  |  |  |
| Miao | 0.579 | 1.784 (0.826-3.405) | 0.105 |  | 1.608 | 4.993 (0.954-17.949) | 0.028* |
| Zhuang | NA | NA | NA |  |  |  |  |
| Buyi | 0.346 | 1.413 (0.226-4.812) | 0.640 |  | 1.487 | 4.423 (0.619-19.058) | 0.076. |
| Korean | NA | NA | NA |  |  |  |  |
| Man | NA | NA | NA |  |  |  |  |
| Dong | NA | NA | NA |  |  |  |  |
| Tujia | NA | NA | NA |  |  |  |  |
| Other | NA | NA | NA |  |  |  |  |
| Highest Level of Education Attained |  |  |  |  |  |  |  |
| None | - | 1.000 | - |  | - | 1.000 | - |
| Grad from primary | -0.249 | 0.780 (0.549-1.104) | 0.161 |  | -0.595 | 0.552 (0.295-1.004) | 0.056. |
| Lower middle school degree | -0.446 | 0.640 (0.451-0.905) | 0.012* |  | -0.161 | 0.852 (0.471-1.529_ | 0.592 |
| Upper middle school degree | -0.318 | 0.728 (0.473-1.098) | 0.137 |  | 0.315 | 1.370 (0.698-2.630) | 0.351 |
| Technical or vocational degree | -0.052 | 0.949 (0.525-1.621) | 0.856 |  | 0.137 | 1.146 (0.439-2.738) | 0.768 |
| University or college degree | -0.122 | 0.885 (0.385-1.777) | 0.751 |  | 0.142 | 1.153 (0.287-3.755) | 0.827 |
| Master's degree or higher | NA | NA | NA |  |  |  |  |
| Smoking recorded yes | -0.202 | 0.817 (0.614-1.077) | 0.160 |  | -0.016 | 0.984 (0.568-1.684) | 0.954 |
| Alcohol recorded yes | -0.365 | 0.694 (0.516-0.922) | 0.014* |  | -0.436 | 0.647 (0.374-1.098) | 0.112 |
| Coffee recorded yes | -0.435 | 0.647 (0.158-1.748) | 0.462 |  |  |  |  |
| Soft drink recorded yes | -0.703 | 0.495 (0.342-0.697) | <0.001*** |  | -0.463 | 0.629 (0.353-1.071) | 0.100 |
| Tea recorded yes | 0.306 | 1.358 (1.055-1.748) | 0.017* |  | -0.145 | 0.865 (0.554-1.341) | 0.520 |
| Hypertension recorded yes | 1.446 | 4.247 (3.153-5.670) | <0.001*** |  | 0.733 | 2.081 (1.203-3.546) | 0.008** |
| Waist circumference (cm) | 0.089 | 1.093 (1.079-1.108) | <0.001*** |  | 0.032 | 1.033 (1.001-1.065) | 0.043* |
| BMI (kg/m²) | 0.227 | 1.255 (1.212-1.299) | <0.001*** |  | 0.033 | 1.034 (0.949-1.126) | 0.450 |
| Triceps skin fold (cm) | 0.032 | 1.033 (1.019-1.046) | <0.001*** |  | 0.008 | 1.008 (0.979-1.035) | 0.594 |
| SBP (mmHg) | 0.025 | 1.025 (1,018-1.033) | <0.001*** |  | 0.007 | 1.007 (0.992-1.022) | 0.360 |
| DBP (mmHg) | 0.035 | 1.035 (1.022-1.049) | <0.001*** |  | -0.003 | 0.997 (0.972-1.023) | 0.830 |
| Sleep time (hours) | 0.008 | 1.009 (0.911-1.114) | 0.865 |  |  |  |  |
| Physical activity |  |  |  |  |  |  |  |
| Very light | - | 1.000 | - |  | - | 1.000 | - |
| Light | -0.186 | 0.830 (0.604-1.141) | 0.250 |  | 0.291 | 1.338 (0.796-2.266) | 0.274 |
| Moderate | -0.804 | 0.448 (0.286-0.681) | <0.001*** |  | -0.163 | 0.849 (0.407-1.704) | 0.653 |
| Heavy | -0.899 | 0.407 (0.284-0.577) | <0.001*** |  | -0.544 | 0.581 (0.299-1.113) | 0.104 |
| Very heavy | -0.899 | 0.407 (0.023-1.947) | 0.380 |  |  |  |  |
| No working ability | -0.250 | 1.284 (0.582-2.526) | 0.499 |  | -0.025 | 0.975 (0.293-2.283) | 0.965 |
| Diet |  |  |  |  |  |  |  |
| Total calories (kcal) | 0.000 | 1.000 | 0.176 |  |  |  |  |
| Carbohydrate (g) | -0.002 | 0.998 (0.996-0.999) | <0.001*** |  | -0.002 | 0.998 (0.995-1.000) | 0.068. |
| Fat (g) | 0.003 | 1.003 (1.000-1.006) | 0.070. |  | 0.003 | 1.003 (0.997-1.008) | 0.321 |
| Protein (g) | 0.003 | 1.003 (0.998-1.007) | 0.259 |  | 0.012 | 1.012 | 0.005** |
| Blood test |  |  |  |  |  |  |  |
| HDL (mmol/L) | -0.903 | 0.405 (0.265-0.610) | <0.001*** |  | -0.274 | 0.761 (0.344-1.524) | 0.490 |
| LDL (mmol/L) | 0.133 | 1.143 (1.006-1.287) | 0.033* |  | -0.052 | 0.949 (0.604-1.361) | 0.843 |
| Insulin (μIU/L） | 0.005 | 1.005 (1.000-1.009) | 0.023* |  | -0.018 | 0.982 (0.964-0.997) | 0.034* |
| HbA1c (%) | 1.177 | 3.246 (2.812-3.788) | <0.001*** |  | 0.503 | 1.654 (1.278-2.160) | <0.001*** |
| Glucose (mmol/L) | 0.804 | 2.234 (2.036-2.465) | <0.001*** |  | 0.514 | 1.672 (1.412-1.986) | <0.001*** |
| TG (mmol/L) | 0.243 | 1.275 (1.181-1.374) | <0.001*** |  | -0.039 | 0.962 (0.757-1.192) | 0.812 |
| TC (mmol/L) | 0.257 | 1.293 (1.139-1.468) | <0.001*** |  | -0.070 | 0.933 (0.453-1.451) | 0.767 |
