# Supplementary material for: Development and validation of a prediction model estimating the 10-year risk for type 2 diabetes in China
Source: PLoS One. 2020 Sep 3;15(9):e0237936. doi: 10.1371/journal.pone.0237936 (PMC7470416; doi:10.1371/journal.pone.0237936)
Supplement: S2 Table — (DOCX) [file pone.0237936.s002.docx]

| S2 Table. Feature selection using the least absolute shrinkage and selection operator (LASSO) model binary logistic regression model. | | | | |
| --- | --- | --- | --- | --- |
|  | model A | model B | model C | model D |
| minimum λ value | 0.000 | 0.001 | 0.002 | 0.001 |
| log (λ) | -8.099 | -6.520 | -6.227 | -6.526 |
| Age (years) | 0.157 | 0.155 | 0.099 |  |
| Gender | -0.488 | -0.276 | exclude |  |
| Ethnic Groups | -0.029 | -0.026 | exclede |  |
| Hypertension recorded yes | 0.624 | 0.575 | 0.522 |  |
| Smoking recorded yes | -0.170 | exclude | exclude |  |
| Alcohol recorded yes | -0.457 | -0.024 | -0.217 |  |
| Waist circumference (cm) | 0.515 | 0.494 | 0.293 |  |
| BMI (kg/m²) | 0.467 | 0.461 | 0.023 |  |
| Highest Level of Education Attained |  | 0.076 | 0.109 |  |
| Coffee recorded yes |  | excluded | exclude |  |
| Soft drink recorded yes |  | -0.491 | -0.258 |  |
| Tea recorded yes |  | 0.046 | -0.024 |  |
| Physical activity |  | -0.024 | -0.105 |  |
| Total calories (kcal) |  | -0.139 | -0.223 |  |
| Carbohydrate (g) |  | -0.130 | -0.042 |  |
| Fat (g) |  | 0.079 | 0.143 |  |
| Protein (g) |  | 0.186 | 0.329 |  |
| Triceps skin fold (cm) |  | 0.002 | 0.099 |  |
| SBP (mmHg) |  | exclude | exclude |  |
| DBP (mmHg) |  | exclude | exclude |  |
| Sleep time (hours) |  | 0.055 |  |  |
| HDL (mmol/L) |  |  | 0.085 |  |
| LDL (mmol/L) |  |  | exclude |  |
| Insulin (μIU/L） |  |  | -0.258 | -0.097 |
| HbA1c (%) |  |  | 0.919 | 1.144 |
| Glucose (mmol/L) |  |  | 1.026 | 1.037 |
| TG (mmol/L) |  |  | 0.081 | 0.133 |
| TC (mmol/L) |  |  | -0.109 | -0.044 |
