# Supplementary material for: Development and validation of a prediction model estimating the 10-year risk for type 2 diabetes in China
Source: PLoS One. 2020 Sep 3;15(9):e0237936. doi: 10.1371/journal.pone.0237936 (PMC7470416; doi:10.1371/journal.pone.0237936)
Supplement: S3 Table — (DOCX) [file pone.0237936.s003.docx]

| S3 Table. Prediction performance of the nomogram for estimating the 10-year risk of T2DM | | | | | | | | | | | |
| --- | --- | --- | --- | --- | --- | --- | --- | --- | --- | --- | --- |
|  | Model A | |  | Model B | |  | Model C | |  | Model D | |
|  | Training cohort | Validation cohort |  | Training cohort | Validation cohort |  | Training cohort | Validation cohort |  | Training cohort | Validation cohort |
| AUC (95%CI) | 0.788 (0.761-0.816) | 0.818 (0.775-0.861) |  | 0.804 (0.776-0.831) | 0.823 (0.780-0.865) |  | 0.904 (0.877-0.931) | 0.915 (0.877-0.953) |  | 0.885 (0.857-0.913) | 0.862 (0.813-0.912) |
| C index (SD) | 0.788 | 0.818 |  | 0.807 | 0.823 |  | 0.905 | 0.915 |  | 0.882 | 0.862 |
| Best cutoff value | -2.803 | -2.629 |  | -2.878 | -2.482 |  | -3.030 | -2.903 |  | -2.510 | -1.967 |
| Sensitivity, % | 74.71 | 77.17 |  | 77.53 | 77.17 |  | 84.05 | 88.06 |  | 73.08 | 67.65 |
| Specificity, % | 71.16 | 76.91 |  | 70.83 | 79.65 |  | 83.01 | 83.73 |  | 92.25 | 94.59 |
| PPV, % | 13.57 | 17.71 |  | 13.95 | 19.67 |  | 24.73 | 28.23 |  | 39.28 | 47.92 |
| NPV, % | 97.89 | 98.13 |  | 98.10 | 98.18 |  | 98.74 | 98.97 |  | 98.04 | 97.54 |
| TPR, % | 74.71 | 77.17 |  | 77.53 | 77.17 |  | 84.05 | 88.06 |  | 73.08 | 67.65 |
| FPR, % | 28.84 | 23.09 |  | 29.17 | 20.35 |  | 16.99 | 16.27 |  | 7.74 | 5.41 |
| TNR, % | 71.16 | 76.91 |  | 70.83 | 79.65 |  | 83.01 | 83.73 |  | 92.25 | 94.59 |
| FNR, % | 25.29 | 22.83 |  | 22.47 | 22.83 |  | 15.95 | 11.94 |  | 26.92 | 32.35 |
| FDR, % | 86.43 | 82.29 |  | 86.05 | 80.33 |  | 75.27 | 71.77 |  | 60.72 | 52.08 |
| Accuracy, % | 71.37 | 76.92 |  | 71.22 | 79.5 |  | 83.07 | 84.02 |  | 91.02 | 92.74 |
| Precision, % | 13.57 | 17.71 |  | 13.95 | 19.67 |  | 24.73 | 28.23 |  | 0.39 | 47.92 |
| Youden Index | 1.46 | 1.54 |  | 1.48 | 1.57 |  | 1.67 | 1.72 |  | 1.65 | 1.62 |
| *AUC* area under curve, *PPV* positive predictive value, *NPV* negative predictive value, PLR positive likelihood ratio, NLR negative likelihood ratio, TPR true positive rate, FPR false positive rate, FNR false negative rate, FDR false discovery rate. 2000 stratified bootstrap | | | | | | | | | | | |
