# Supplementary material for: Development and validation of a prediction model estimating the 10-year risk for type 2 diabetes in China
Source: PLoS One. 2020 Sep 3;15(9):e0237936. doi: 10.1371/journal.pone.0237936 (PMC7470416; doi:10.1371/journal.pone.0237936)
Supplement: S4 Table — (DOCX) [file pone.0237936.s004.docx]

| S4 Table. Delong comparison between different logistic models | | | | |
| --- | --- | --- | --- | --- |
|  | Training set | | Validation set | |
|  | Z statistic | P value | Z statistic | P value |
| Model A~B. ROC | -0.935 | 0.350 | -0.151 | 0.880 |
| Model A~C. ROC | -6.005 | <0.001^***^ | -3.336 | <0.001^***^ |
| Model A~D. ROC | -4.598 | <0.001^***^ | -1.321 | 0.187 |
| Model B~C. ROC | -5.065 | <0.001^***^ | -3.200 | 0.001** |
| Model B~D. ROC | -3.700 | <0.001^***^ | -1.189 | 0.235 |
| Model C~D. ROC | 1.140 | 0.254 | 1.655 | 0.098 |
