# Supplementary material for: Development and validation of a prediction model estimating the 10-year risk for type 2 diabetes in China
Source: PLoS One. 2020 Sep 3;15(9):e0237936. doi: 10.1371/journal.pone.0237936 (PMC7470416; doi:10.1371/journal.pone.0237936)
Supplement: S5 Table — Data are presented as median (interquartile range), mean (SD), number (%). (DOCX) [file pone.0237936.s005.docx]

| S5 Table. baseline characters of the participants inclued and excluded | | | |
| --- | --- | --- | --- |
| Characteristics | inclued participants(N=6023) | excluded participants (N=5453) | P value |
| Age, mean (SD, yrs) | 48.3 (13.7) | 32.3 (21.0) | <0.001*** |
| Gender |  |  |  |
| Male | 2802 (46.5) | 2833 (51.2) | <0.001*** |
| Female | 3221 (53.5) | 2699 (48.8) |  |
| Ethnic Groups |  |  |  |
| Han | 5526 (91.7) | 4551 (82.3) | <0.001*** |
| Mongolian | 12 (0.2) | 12 (0.2) |  |
| Hui | 17 (0.3) | 21 (0.4) |  |
| Miao | 91 (1.5) | 245 (4.4) |  |
| Zhuang | 59 (1.0) | 47 (0.8) |  |
| Buyi | 25 (0.4) | 300 (5.4) |  |
| Korean | 7 (0.1) | 3 (0.1) |  |
| Man | 217 (3.6) | 130 (2.4) |  |
| Dong | 2 (0.03) | 2 (0.03) |  |
| Tujia | 12 (0.2) | 174(3.1) |  |
| Other | 55 (0.9) | 42 (0.8) |  |
| Highest Level of Education Attained |  |  |  |
| None | 1199 (19.9) | 1426 (25.8) | 0.798 |
| Grad from primary | 1543 (25.6) | 1212 (21.9) |  |
| Lower middle school degree | 1851 (30.7) | 1829 (33.1) |  |
| Upper middle school degree | 819 (13.6) | 619 (11.2) |  |
| Technical or vocational degree | 373 (6.2) | 287 (5.2) |  |
| University or college degree | 237 (3.9) | 154 (2.8) |  |
| Master's degree or higher | 1 (0.02) | 1 (0.02) |  |
| Smoking recorded | 1956 (32.5) | 1265 (22.9) | <0.001*** |
| Alcohol recorded | 1138 (18.9) | 759 (13.7) | 0.108 |
| Coffee | 93 (1.5) | 137 (2.5) | <0.001*** |
| Soft drink | 1421 (23.6) | 2330 (42.1) | <0.001*** |
| Tea | 2215 (36.8) | 1610 (29.1) | 0.001 |
| Hypertension | 582 (9.7) | 293 (5.3) | <0.001*** |
| Waist circumference, mean (SD, cm) | 81.7 (9.8) | 74.0 (12.8) | <0.001*** |
| BMI, mean (SD, kg/m²) | 23.3 (3.7) | 21.0 (3.9) | <0.001*** |
| Triceps skin fold, mean (SD,mm) | 15.7 (8.4) | 13.5 (8.0) | <0.001*** |
| sistolic pressure, mean (SD,mmHg) | 123.4 (18.7) | 114.4 (19.2) | <0.001*** |
| diastolic pressure, mean (SD, mmHg) | 79.4 (11.3) | 74.3 (11.9) | <0.001*** |
| sleep time, mean (SD, hours) | 7.7 (2.9) | 8.0 (3.2) | <0.001*** |
| Physical activity |  |  |  |
| Very light | 1392 (23.1) | 787 (14.2) | <0.001*** |
| Light | 1417 (23.5) | 792 (14.3) |  |
| Moderate | 926 (15.4) | 2032 (36.7) |  |
| Heavy | 2086 (34.6) | 1392 (25.2) |  |
| Very heavy | 34 (0.6) | 16 (0.3) |  |
| Diet |  |  |  |
| Total calories, median (interquartile range, kcal) | 2139.3 (1736.8 - 2601.7) | 1999.8 (1589.3 - 2481.4) | <0.001*** |
| Carbohydrate, median (interquartile range, g) | 306.1 (241.3 - 376.9) | 296.5 (230.2 - 376.6) | <0.001*** |
| Fat, mean (interquartile range, g) | 65.7 (44.8 - 91.6) | 56.6 (35.9 - 82.3) | <0.001*** |
| Protein, mean (interquartile range, g) | 64.1 (50.4 - 79.8) | 59.4 (45.8 - 75.3) | <0.001*** |
| Blood test |  |  |  |
| HDL, mean (SD, mmol/L) | 1.4 (0.5) | 1.4 (0.4) | <0.001*** |
| LDL, mean (SD, mmol/L) | 3.1 (1.0) | 2.7 (0.9) | <0.001*** |
| insulin, median (interquartile range, μIU/L） | 10.2 (7.2 - 14.9) | 10.6 (7.2 - 16.3) | 0.027 |
| HbA1c, mean (SD, %) | 5.7 (1.0) | 5.5 (0.8) | <0.001*** |
| Glucose, mean (SD, mmol/L) | 5.5 (1.5) | 5.3 (1.3) | <0.001*** |
| TG, mean (SD, mmol/L) | 1.7 (1.4) | 1.5 (1.4) | <0.001*** |
| TC, mean (SD, mmol/L) | 5.0 (1.0) | 4.6 (1.0) | <0.001*** |
